# Phthalates and bone mineral density: a systematic review

**DOI:** 10.1186/s12940-022-00920-5

**Published:** 2022-11-12

**Authors:** Nina Z. Heilmann, Katherine W. Reeves, Susan E. Hankinson

**Affiliations:** Department of Biostatistics and Epidemiology, University of Massachusetts Amherst, Amherst, MA 01003 USA

**Keywords:** Phthalates, Bone mineral density, Biomarkers, Review

## Abstract

**Background:**

Exposure to endocrine disruptors, such as phthalates, may impact bone mineral density (BMD) through a variety of mechanisms. Studies of phthalate exposure and BMD in humans are scarce.

**Objectives:**

To synthesize published data on the association between phthalate metabolites and BMD in humans and to provide methodological suggestions for future research.

**Methods:**

A single investigator searched PubMed for relevant studies, including observational studies of phthalate exposure and BMD in children and postmenopausal women. Twelve studies were screened with 5 meeting the eligibility criteria and included for review. A quality assessment form was used as a quality measure and key information was extracted from the included studies.

**Results:**

In one prospective study among postmenopausal women, higher levels of monocarboxyoctyl phthalate (MCOP) and monocarboxynonyl phthalate (MCNP) were significantly associated with lower BMD among nonusers of hormone therapy (HT). In cross-sectional studies of postmenopausal women, monoethyl phthalate (MEP), mono-n-butyl phthalate (MnBP), mono (3-carboxypropyl) phthalate (MCPP), and mono-benzyl phthalate (MBzP) were negatively associated with BMD, and MCNP was positively associated with BMD, but these results were not replicated across studies. In studies of fetal exposure to phthalates and childhood BMD, significant positive associations between MCPP and BMD in children at age 12 years were found in 1 study, while associations were null in the other study.

**Conclusions:**

Studies among postmenopausal women provide suggestive evidence of an association between urinary phthalate metabolite concentration and decreased BMD. Results from studies of childhood BMD are inconclusive given the limited data and their limitations. More research is needed to address limitations and further investigate the association between phthalate exposure and human BMD.

**Supplementary Information:**

The online version contains supplementary material available at 10.1186/s12940-022-00920-5.

## Introduction

Bone mineral density (BMD) is a measure of bone health and is strongly related to fracture risk. BMD declines rapidly with age, leaving older adults most susceptible to fractures [1]. Typically, BMD is represented either absolutely (in g/cm^2^), or by a Z-score, which compares an individual’s BMD to the average values for a healthy person of the same age and sex [2]. Osteoporosis, a disease characterized by bone loss and deterioration of bone structure, is the leading cause of fractures in the U.S. and affects almost 20% of women and 5% of men aged 50 and over [1, 3]. An estimated 6.3 million bone fractures occur every year in the U.S. [1], with the highest fracture rates seen in youth and old age [4]. Throughout childhood and adolescence, just under 20% of children experience a fracture requiring an emergency department visit [5]. Fractures are associated with high morbidity, mortality, and medical costs, and are projected to increase globally because of the aging of the population [6]. While behavioral risk factors for low BMD (e.g., smoking, low physical activity, and poor diet) are well established [1], studies on environmental determinants of low BMD are scarce.

Phthalates are a group of chemicals used as plasticizers in a variety of products (e.g., toys, cosmetics, vinyl flooring, and personal care products). Phthalate exposure is widespread, as measurable levels of many urinary phthalate metabolites have been detected in nearly all U.S. National Health and Examination Survey participants (NHANES) [7]. Exposure can occur from ingestion, inhalation, and dermal contact with products that contain phthalates [8].

Phthalates are well-established endocrine disrupting chemicals (EDCs), and research suggests that EDCs have the potential to disrupt bone homeostasis and hormonal regulation of the bone remodeling process [9]. A number of biological mechanisms have been hypothesized to explain the ways phthalate metabolites may alter bone processes, but studies investigating the association between phthalates and BMD in human populations are limited. Recent animal studies provide experimental evidence of a link between phthalate exposure and inhibitory effects on healthy bone processes and homeostasis in rodents. Two experimental studies found that adult male mice exposed to low dibutyl phthalate (DBP) doses prepubertally [10] and to di(2-ethylhexyl) phthalate (DEHP) in adulthood [11] had lower BMD and altered bone microstructure. Additional studies determined that BMD was significantly reduced in ovariectomized mice treated with high doses of DEHP [12] and in intact mice injected with diisononyl phthalate (DINP) [13]. Certain phthalates have also been observed to damage DNA, increase the number of skeletal malformations, and induce osteoblast apoptosis in rodents, thereby affecting BMD [14].

Existing knowledge and research contribute to the biologic plausibility of an effect of phthalate exposure on human BMD in older adults. Several studies have found phthalate metabolite concentrations to be positively associated with oxidative stress and inflammation, both of which may reduce BMD in older adults [15–18]. Recent studies have also suggested adverse effects of certain phthalates, namely DEHP [19, 20] and mono-(3-carboxypropyl) phthalate (MCPP) [20] on vitamin D levels in adults, which has been consistently positively associated with BMD in large studies [21–23].

Childhood and adolescence may be important windows of susceptibility to EDCs in relation to bone health outcomes, as growth and sex hormones play critical roles during this period of rapid skeletal growth [1]. Compared to adults, infants and children may be particularly sensitive to EDCs due to rapid development, greater exposure to EDCs (e.g., high ventilation rates, intestinal absorption, and hand-to-mouth activity), and other differences in anatomy, physiology, and toxicokinetics [24]. Estrogen acts to inhibit bone breakdown at all stages of life and stimulate bone formation. Testosterone stimulates muscle growth, which also increases bone formation [1]. Endocrine disturbances, including vitamin D, growth hormone, and sex hormone deficiencies, have been associated with increased fracture risk and osteoporosis in children [4, 25]. Peak bone mass (PBM), defined as the amount of bone present in the skeleton at the end of its maturation process [25], is considered an important predictor of osteoporosis in adulthood and is influenced by hormonal factors [4, 25]. Approximately 90% of the PBM is acquired before age 20 [4], emphasizing adolescence as a critical period for bone health.

Since phthalates may affect BMD through a variety of pathways, it is important to understand the association between phthalates and BMD in human studies. The aim of this review is to synthesize published data on the association between phthalate biomarker concentrations and BMD in humans and to provide methodological suggestions for future research.

## Methods

A search was performed in PubMed in December 2021 using key words “phthalate” AND “bone mineral density” OR “fracture”. Eligibility criteria included studies that: 1) were observational (i.e., cross-sectional, case-control, or cohort studies); 2) examined the association of phthalate metabolites and bone mineral density in human populations; and 3) were written in English. The results of the search were individually evaluated for relevance and eligibility for this review by a single investigator. Reference lists from included studies were also screened to identify additional relevant publications.

Studies were individually evaluated for quality and risk of biases using a quality assessment form (see Additional file 1: supplemental materials). Key information and study characteristics were extracted from each study, including study design, geographic location, sample size and population characteristics, exposure and outcome measurements, and strength of findings (Table 1). Results presented as “Key Findings” in Table 1 were considered statistically significant at the alpha level of 0.05. Methods of subject selection, exposure and outcome assessment, and statistical analyses were analyzed for risk of bias and error.

**Table 1:** Studies Examining Urinary Phthalate Metabolites and Bone Mineral Density

Author, Publication Year	Study Design	Study Population	Phthalate Metabolites Measured	Outcome (Parameterization)	Key Findings
DeFlorio-Barker et al., 2016 [26]	Cross-sectional	480 postmenopausal women from NHANES 2005-2010 cohort	MEP, MnBP, MiBP, MBzP, MCNP, MCOP, MCPP, MEHP, MEHHP, MEOHP, MECPP, Σ-LMW phthalate metabolites^a^, Σ-DEHP metabolites^b^, Σ-HMW phthalate metabolites^c^, Σ-Anti-androgenic phthalate metabolites^d^, Σ-Estrogenic phthalate metabolites^e^	BMD (g/cm^2^) measured using DXA scans of the lumbar spine, proximal femur, and femoral neck	Associations of phthalate metabolites (continuous) with BMD: Total spine: MEP (β = − 0.038, *p* = 0.001) Σ-LMW phthalate metabolites (β = − 0.044, *p* = 0.003) Σ-Estrogenic phthalate metabolites (β = − 0.046, *p* = 0.002) Femoral neck: MCNP (β = 0.024, *p* = 0.05)
Min & Min, 2014 [27]	Cross-sectional	398 postmenopausal women from NHANES 2005-2008 cohort	MnBP, MCPP, MCNP, MCOP, MECPP, MEP, MBzP, MiBP, Σ-DEHP metabolites (MEHP, MEHHP, MEOHP)	BMD (g/cm^2^) measured using DXA scans of the total hip and femoral neck	Associations of phthalate metabolites with BMD: Total hip: MnBP (β = − 0.048, *p* = 0.035) (Q4 vs. Q1) MCPP (β = − 0.055, *p* = 0.005) (Q4 vs. Q1) MBzP (β = − 0.054, *p* = 0.003) (Q3 vs. Q1) Femoral neck: MnBP (β = − 0.040, *p* = 0.046) (Q4 vs. Q1) MCPP (β = − 0.045, *p* = 0.041) (Q4 vs. Q1) MBzP (β = − 0.041, *p* = 0.024) (Q3 vs. Q1)
Reeves et al., 2021 [28]	Cross-sectional & Prospective Cohort	1255 postmenopausal women from the WHI cohort	MEP, MBzP, MCOP, MCNP, MCPP, Σ-DBP^f^, Σ-DiBP^g^, Σ-DEHP^b^,	BMD (g/cm^2^) measured using DXA scans of the total hip and femoral neck	Longitudinal associations between phthalate biomarkers and 3-year percentage change BMD measures among non-HT users: Total hip: MCOP (β = − 1.80, 95% CI − 2.81 to − 0.78) (Q4 vs. Q1) MCNP (β = − 1.84, 95% CI − 2.80 to − 0.89) (Q4 vs. Q1) Femoral neck: MCNP (β = − 1.53, 95% CI − 2.75 to − 0.31) (Q4 vs. Q1)
Van Zwol-Janssens et al., 2020 [29]	Prospective cohort	1362 mother-child pairs from the Generation R Study	Σ-LMW phthalate metabolites (MMP, MEP, MiBP, MnBP), Σ-HMW phthalate metabolites (MBzP, MHP, mono-2-heptyl phthalate), Σ-DEHP (MECPP, MCMHP, MEHHP, MEOHP), Σ-DNOP (MCPP)	BMD (mg/cm^2^) measured using DXA scans of the whole body (excluding head)	Adjusted associations of maternal phthalate concentrations with childhood total body BMD at age 6 years: Σ-LMW phthalate metabolites: First trimester (β = − 0.61, 95% CI − 2.89 to 1.67) Second trimester (β = 0.92, 95% -1.44 to 3.29) Third trimester (β = 1.81, 95% -0.69 to 4.30) Σ-DEHP metabolites: First trimester (β = − 0.41, 95% CI − 2.45 to 1.63) Second trimester (β = 0.13, 95% -1.90 to 2.17) Third trimester (β = 0.30, 95% -1.82 to 2.41) Σ-HMW (high-molecular-weight) phthalate metabolites: First trimester (β = − 0.67, 95% CI − 2.74 to 1.41) Second trimester (β = 0.56, 95% CI − 1.46 to 2.58) Third trimester (β = − 0.15, 95% CI − 1.98 to 2.28) Σ-DNOP metabolites: First trimester (β = − 1.48, 95% CI − 3.43 to 0.47) Second trimester (β = 1.19, 95% CI − 0.95 to 3.34) Third trimester (β = − 1.39, 95% CI − 3.45 to 0.68)
Kuiper et al., 2022 [30]	Prospective cohort	233 mother-child pairs from the HOME Study	MEP, MiBP, MnBP, MBzP, MCPP, Σ-DEHP^b^	BMD and BMAD (height-for-age adjusted age-, sex-, and population ancestry-specific Z-scores) measured using DXA scans of the whole body (excluding head), lumbar spine, total hip, femoral neck, 1/3rd distal radius, and ultradistal radius	Adjusted mean differences (β and 95% Cis) in BMD Z-score at age 12 associated with a doubling in prenatal phthalate biomarkers: Whole body (excluding head): MEP (β = 0.11, 95% CI 0.04 to 0.18) in overall sample MEP (β = 0.15, 95% CI 0.06 to 0.24) in males Total hip: MEP (β = 0.09, 95% CI 0.01 to 0.18) in overall sample MEP (β = 0.13, 95% CI 0.01 to 0.24) in males Femoral neck: MEP (β = 0.13, 95% CI 0.01 to 0.25) in males 1/3rd Distal radius: MEP (β = 0.09, 95% CI 0.01 to 0.17) in overall sample MCPP (β = 0.16, 95% CI 0.01 to 0.31) in overall sample Ultradistal radius: MBzP (β = − 0.10, 95% CI − 0.24 to 0.03) in males MBzP (β = 0.16, 95% CI − 0.03 to 0.35) in females *P* interaction = 0.03 MCPP (β = 0.21, 95% CI 0.04 to 0.38) in overall sample MCPP (β = − 0.05, 95% CI − 0.27 to 0.17) in males MCPP (β = 0.41, 95% CI 0.17 to 0.64) in females *P* interaction = 0.01 Spine BMAD: MEP (β = 0.15, 95% CI 0.05 to 0.25) in males MEP (β = − 0.01, 95% CI − 0.13 to 0.12) in females *P* interaction = 0.05 MiBP (β = 0.21, 95% CI 0.04 to 0.39) in females Adjusted mean differences (β and 95% CrIs) in aBMD Z-scores at age 12 associated with the 90th percentile of the maternal urinary phthalate mixture compared to the 50th percentile: 1/3rd distal radius: (β = 0.52, 95% CrI 0.09 to 0.96) Ultradistal radius: (β = 0.51, 95% CrI 0.03 to 0.98) Total hip: (β = 0.50, 95% CrI 0.04 to 0.96)

Abbreviations: *BMAD*: bone mineral apparent density, *BMD*: bone mineral density, *BMI*: body mass index, *DBP*: dibutyl phthalate, *DEHP*: di(2-ethylhexyl) phthalate, *DiBP*: di-isobutyl phthalate, *DINP*: diisononyl phthalate, *DNOP*: di-n-octyl phthalate, *DXA*: dual-energy X-ray absorptiometry, *HMW*: high-molecular-weight, *HOME*: Health Outcomes and Measures of the Environment, *HT*: hormone therapy, *LMW*: low-molecular-weight, *MBP*: mono-n-butyl phthalate, *MBzP*: mono-benzyl phthalate, *MCMHP*: mono (2-carboxymethylhexyl) phthalate, *MCNP*: monocarboxynonyl phthalate, *MCOP*: monocarboxyoctyl phthalate, *MCPP*: mono (3-carboxypropyl) phthalate, *MECPP*: mono (2-ethyl-5-carboxypentyl) phthalate, *MEHP*: mono (2-ethylhexyl) phthalate, *MEHHP*: mono (2-ethyl-5-hydroxyhexyl) phthalate, *MEOHP*: mono (2-ethyl-5-oxohexyl) phthalate, *MEP*: monoethyl phthalate, *MHP*: mono-n-hexyl phthalate, *MHBP*: monohydroxybutyl phthalate, *MHiBP*: mono-hydroxyisobutyl phthalate, *MiBP*: monoisobutyl phthalate, *MMP*: monomethyl phthalate, MnBP: mono-n-butyl phthalate, *NHANES*: National Health and Nutrition Examination Survey, *WHI*: Women’s Health Initiative

Notes: All phthalate metabolites were measured using high-performance liquid chromatography. Results presented were considered statistically significant at the alpha level of 0.05

^a^Molar sum of MEP, MnBP, MiBP, expressed as MEP

^b^Molar sum of MEHP, MEHHP, MEOHP, MECPP, expressed as MEHP

^c^Molar sum of MBzP, MnBP, MCNP, MCOP, MCPP, MEHP, MEHHP, MEOHP, MECPP, expressed as MEHP

^d^Molar sum of anti-androgenic metabolites (MBzP, MnBP, MEHP, MEHHP, MEOHP, MECPP), expressed as MEHP

^e^Molar sum of estrogenic metabolites (MEP, MnBP, MiBP, MBzP), expressed as MEP

^f^Molar sum of MBP, MHBP

^g^Molar sum of MiBP, MHiBP

## Results

**Fig. 1:**
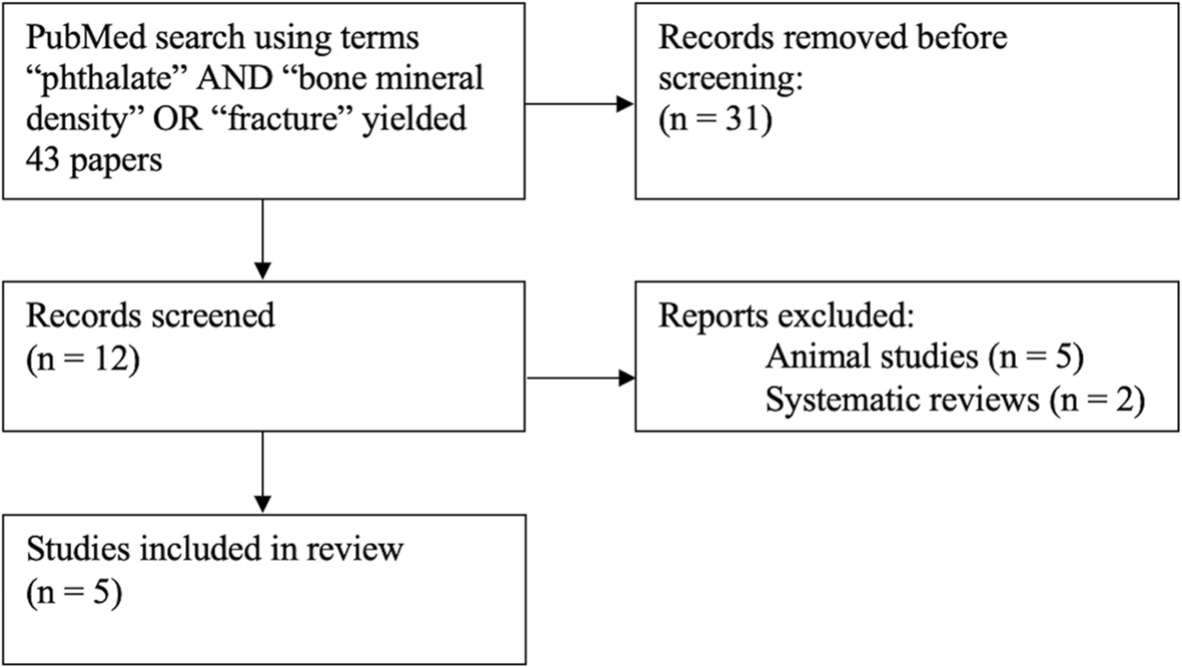
Identification of studies via databases

The initial search in PubMed using the combination “phthalate” AND “bone mineral density” OR “fracture” yielded 43 results. Twelve articles that focused on the association of urinary phthalate metabolites and bone health were screened for eligibility. Seven studies were excluded because they were either animal studies (5 studies) or systematic reviews (2 studies) (Fig. 1). Of the 5 included studies, 2 were cross-sectional [26, 27] and 3 were prospective cohort studies [28–30]. Two cross-sectional studies and 1 prospective cohort study included postmenopausal women aged 50 years and older, while 2 prospective cohort studies followed participants from gestation to early childhood.

Two cross-sectional studies obtained data from NHANES. One study used the 2005-2006 and 2007-2008 NHANES with a total sample size of 398 women [27]. The other study used continuous NHANES from 2005 to 2010 with 480 total participants [26]. Women were considered postmenopausal if they reported not having a menstrual period in the previous 12 months or indicated surgical menopause, such as bilateral oophorectomy. Phthalates were measured in urine samples which were processed using enzyme deconjugation of the glucuronidated phthalate monoester metabolites with high-performance liquid chromatography (HPLC)-electrospray ionization mass spectrometry. BMD measurements were obtained with dual-energy X-ray absorptiometry (DXA) scans of the spine [26], hip [27], and femur [26, 27]. Significant associations between urinary phthalate metabolites and BMD were observed in both studies. DeFlorio-Barker et al. observed statistically significant negative associations between total spine BMD and monoethyl phthalate (MEP) (β = − 0.038, *p* = 0.001), the molar sum of all low-molecular-weight (LMW) metabolites (mono-n-butyl phthalate [MnBP], monoisobutyl phthalate [MiBP], MEP) (β = − 0.044, *p* = 0.003), and the molar sum of estrogenic metabolites (β = − 0.046, *p* = 0.002) and observed a significant positive association between monocarboxynonyl phthalate (MCNP) and femoral neck BMD (β = 0.024, *p* = 0.05) [26]. Min & Min found significant inverse associations of femoral neck BMD with MnBP (β = − 0.040, *p* = 0.046 for Q4 vs. Q1) and MCPP levels (β = − 0.045, *p* = 0.041 for Q4 vs. Q1). Additionally, women in the high MnBP (β = − 0.048, *p* = 0.035 for Q4 vs. Q1), MCPP (β = − 0.055, *p* = 0.005 for Q4 vs. Q1), and monobenzyl phthalate (MBzP) (β = − 0.054, *p* = 0.003 for Q3 vs. Q1) quartiles had significantly lower BMDs than those in the lowest quartile (Q1), for total hip BMD [27] A strength of these studies is that they utilized data from NHANES, which is nationally representative and generalizable to the U.S. population. However, causality and temporality cannot be drawn given the cross-sectional nature of these studies. Exposure misclassification must also be considered, as phthalate metabolites metabolize quickly and were only measured in single spot urine samples. Due to high day-to-day and time-of-day variability of phthalate metabolite concentrations within individuals, a single sample may over- or underestimate long-term exposure and bias effect estimates in larger populations toward the null [31].

The largest study evaluating the association between urinary phthalate metabolites and BMD among postmenopausal women was conducted among a sample of 1255 participants in the Women’s Health Initiative (WHI) [28]. Urine samples were provided by participants at their baseline and year 3 clinic visits. Urinary phthalate metabolites were measured using enzymatic deconjugation and HPLC-electrospray ionization tandem mass spectrometry. BMD was measured at the total hip and femoral neck using DXA at baseline, year 3, and year 6 clinic visits. Cross-sectional and longitudinal associations between phthalate biomarkers and BMD were assessed, with stratification by hormone therapy (HT) use. Longitudinal analyses estimated associations of phthalate biomarkers with percent change in BMD over a 3-year interval. Cross-sectional analyses found significant inverse associations in HT nonusers between MCPP and BMD at the hip (β = − 8.22, 95% CI − 14.19 to − 2.24 for Q3 vs. Q1) and femoral neck (β = − 11.22, 95% CI − 18.31 to − 4.12 for Q3 vs. Q1), along with lower total hip BMD among women with high urinary concentrations of ΣDiBP (the sum of metabolites of di-isobutyl phthalate) (β = − 11.09, 95% CI − 18.69 to − 3.49 for Q4 vs. Q1). In longitudinal analyses, higher urinary concentrations of monocarboxyoctyl phthalate (MCOP) (β = − 1.80, 95% CI − 2.81 to − 0.78 for Q4 vs. Q1) and MCNP (− 1.84, 95% CI − 2.80 to − 0.89 for Q4 vs. Q1) were associated with lower total hip BMD, and MCNP was associated with lower femoral neck BMD among HT nonusers (β = − 1.53, 95% CI − 2.75 to − 0.31 for Q4 vs. Q1). No significant associations were found in cross-sectional or longitudinal analyses among HT users. One limitation of this study was that the sample was highly selected and was not representative of the U.S. population (e.g., 82% of women identified as non-Hispanic White). The issue of temporal variability of phthalate metabolites is relevant in this study as well, although each participant provided at least 2 urine samples during the study period to better represent longer-term exposure. Strengths of this study include its prospective design, large size, stratification by HT use, and repeated measures of phthalate metabolites and BMD over the study period.

Two prospective cohort studies examined the relationship between fetal phthalate exposure and BMD in early adolescence. One study selected a sample of 223 mother-child pairs from the Health Outcomes and Measures of the Environment (HOME) study in Cincinnati, Ohio [30]. Pregnant women were recruited from 2003 to 2006 and their children were prospectively followed until age 12 years. Maternal phthalate biomarkers were quantified in spot urine samples collected at 16 and 26 weeks of gestation and were analyzed by isotope dilution HPLC coupled with tandem mass spectrometry [32]. At the age 12 study visit, BMD was measured for the whole body, femoral neck, radius, total hip, and spine using DXA. Significant positive associations between 16- and 26- week averaged concentrations of MEP and MCPP with BMD at age 12 for nearly all bone sites was found. For example, a doubling in average urinary MCPP concentration was associated with a 0.21 greater ultradistal radius areal BMD (aBMD) Z-score (β = 0.21, 95% CI 0.04 to 0.38). A 2-fold increase in MEP concentration was associated with a 0.11 greater aBMD score for the whole body (excluding head) aBMD Z-score. Child’s sex modified the associations of certain phthalate biomarkers at various bone sites, although the direction of the effect was inconsistent. For example, a significant positive association of MEP and spine bone mineral apparent density (BMAD) was observed for males (β = 0.15, 95% CI 0.05 to 0.25), while a non-significant negative association was found in females (β = − 0.01, 95% CI − 0.13 to 0.12; *P* interaction = 0.05). Effect modification by sex was also observed for MBzP (*P* interaction = 0.03) and MCPP (*P* interaction = 0.01) at the ultradistal radius, although the direction of this effect was reversed (i.e., non-significant negative associations were found in males [MBzP: β = − 0.10, 95% CI − 0.24 to 0.03; MCPP: β = − 0.05, 95% CI − 0.27 to 0.17] and positive associations were observed among females [MBzP: β = 0.16, 95% CI − 0.03 to 0.35; MCPP: β = 0.41, 95% CI 0.17 to 0.64]). Stratified analyses by sex may have been underpowered to detect all associations due to the moderate sample size. Along with analyzing associations of individual phthalate metabolites with BMD, Kuiper et al. investigated associations of mixtures of all measured metabolites with BMD using Bayesian kernel machine regression (BKMR) and quantile g-computation. In BKMR models, when holding phthalate metabolites at the 90th percentile vs. the 50th percentile, associations between phthalate biomarker mixtures and aBMD were strongest for the 1/3rd distal radius (β = 0.52, 95% credible interval (CrI) 0.09 to 0.96), ultradistal radius (β = 0.51, 95% CrI 0.03 to 0.98), and total hip (β = 0.50, 95% CrI 0.04 to 0.96), suggesting a positive association between exposure to phthalates and BMD. Phthalate exposure may be misclassified, as maternal urinary phthalate metabolite concentrations during pregnancy may not be representative of true fetal concentrations of phthalates. This misclassification would tend to bias effect estimates toward the null, as a mother’s urinary level may be an over- or underestimate of her child’s true phthalate exposure and the misclassification is unlikely to be related to the child’s BMD. Strengths of this study were that associations of phthalates and BMD were measured prospectively and BMD was measured at multiple bone sites.

A larger prospective cohort study similarly analyzed associations of fetal exposure to phthalates with BMD in school-aged children [29]. A sample of 1362 mother-child pairs from the population-based Generation R Study in Rotterdam, Netherlands was recruited between February 2004 and July 2005. Phthalate concentrations were measured in a spot urine sample collected from participants during the first, second, and third trimester of pregnancy and were quantified using a solid-phase extraction method performed by HPLC-electrospray ionization tandem mass spectrometry. Total body BMD of children was measured at both 6 and 10 years using DXA. No significant associations between maternal phthalate concentrations and childhood BMD were found. The population-based cohort design and large sample size were strengths of this study, along with the repeated measures of maternal urinary phthalate metabolite concentrations at multiple time points during pregnancy. Selection bias is a concern in this study, as significant differences in maternal phthalate urinary concentrations, age, education, and lifestyle factors were reported between those included and not included in the study. Residual confounding due to unmeasured lifestyle variables, such as diet, drug use, and physical activity, may also be an issue.

## Discussion

Significant associations between phthalate metabolite concentrations and BMD were found in all the included studies, although the direction of effects was inconsistent. Higher levels of phthalate metabolites in postmenopausal women were inversely associated with BMD. While individual studies observed negative associations for greater MEP, MnBP, MCPP, MBzP, and MCOP concentrations with lower BMD, these findings were not replicated in the other studies. Interestingly, MCNP was significantly positively associated with BMD in one study [26], but significantly negatively associated with BMD in another [28]. Inconsistent results were likely attributable to differing study designs, covariates considered, and statistical analyses performed. Reeves et al. were the first to evaluate the association in postmenopausal women prospectively and with stratification by HT use. In cross-sectional studies, Min & Min did not stratify by hormone use but controlled for it in their models, while DeFlorio-Barker et al. excluded women who were taking hormone medications altogether. Overall, the 3 studies provide suggestive evidence of an association between urinary phthalate metabolite concentration and lower BMD in postmenopausal women aged 50 or older.

In the 2 studies of fetal exposure to phthalates and childhood BMD, the effect of phthalates on BMD was less clear. Kuiper et al. found significant positive associations between MCPP and BMD in children at 12 years in males and females in a smaller study. Sex modified the association for MBzP and MCPP at the ultradistal radius and for MEP at the spine [30]. In the larger study, van Zwol – Janssens et al. had overall null results with no sex-specific associations. Differences in covariates considered, quantification of BMD (i.e., raw values vs. Z-scores), sample size, and measurement errors may contribute to the inconsistent results found between studies. The use of maternal urinary phthalate metabolite concentrations as a proxy to fetal phthalate exposure, along with the high temporal variability of phthalate metabolites, likely led to exposure misclassification and muddied associations. In consideration of these limitations, results should only be used to generate hypotheses for further investigations of the effect of phthalates on BMD in children.

Several phthalates were associated with greater BMD at multiple skeletal sites in children, but it is unclear what the long-term impact is on bone health throughout the life course. BMD is not an inherent predictor of bone health and fracture risk. Rather, a combination of other factors, including nutrition, physical activity, and timing of skeletal development, puberty, and attainment of peak bone mass, contribute to bone strength and health [33]. Further research is required to elucidate the array of bone measures that impact positive bone health throughout the life span and whether peak bone mass itself, the timing of bone mass accrual, or other indicators of bone health during adolescence are strong predictors of fracture risk in later life.

All studies used DXA to measure BMD, which is recognized by the World Health Organization as the best technique for assessing BMD in postmenopausal women [34]. DXA scans were taken at various skeletal sites, including the lumbar spine, femoral neck, proximal femur, total hip, forearm, and whole body. The 3 studies on postmenopausal women measured femoral neck and total hip BMD, which are considered the optimal sites for predicting fracture risk in older adults [34]. The 2 studies on children performed lumbar spine and whole body scans, which are recognized as the best skeletal sites for assessing BMD in children and adolescents [35]. Variability in outcome assessment may have occurred through technician variability, device errors, or skeletal site differences. However, all studies reported the utilization of various quality assurance and calibration methods, so the effect of outcome misclassification was likely minimal.

The skeletal sites assessed across the studies include both cortical and trabecular bone sites. Cortical bone is the dense outer surface of bone that makes up about 75-80% of the total skeletal mass, while trabecular bone is a spongy, porous form of bone tissue that makes up the other 20-25% [1]. Bone turnover and bone loss occur at different rates in trabecular compared to cortical bone, and each compartment is differentially affected by hormones between males and females and with age [36]. Two studies investigated the association of phthalate metabolites with BMD measured at the lumbar spine, which is rich in trabecular bone [36]. Higher MEP level was significantly associated with lower lumbar spine BMD in one study of postmenopausal women [26], and higher lumbar spine BMD in only males in one study of children [30]. No other measured phthalate metabolites were associated with BMD at sites that are more predominantly trabecular bone sites across studies. Other skeletal sites assessed in the included studies were cortical bone sites, including the hip, femoral neck, and radius [36]. There were no consistent, significant associations between phthalate metabolites and these sites that are predominantly cortical bone. When grouping cortical sites together, MCPP was significantly negatively associated with BMD at cortical sites in one study of postmenopausal women [27], and significantly positively associated with BMD at cortical sites in one study of children [30]. When grouping bone sites by cortical and trabecular bone, there were no consistent (i.e., same direction), significant associations between specific phthalates and BMD across studies. However, DXA can only provide information on areal BMD and cannot differentiate between cortical and trabecular compartments [37]. Inference cannot be made about whether certain phthalates differentially impact cortical versus trabecular bone based on the included studies, although it is plausible due to the different structural and physiological functions of each compartment [36].

Although DXA is the clinical standard for assessing BMD and determining fracture risk, it does not provide information about bone microstructure [37]. Other techniques, such as high-resolution peripheral quantitative computed tomography (HR-pQCT), exist that can measure bone morphology, microarchitecture, and cortical and trabecular compartments separately and with lower radiation exposure than central QCT [38]. A recent study of adult men and women found that cortical and trabecular bone microarchitecture and density measured by HR-pQCT predicted fracture risk independently from BMD assessed by DXA. Future studies could use a combination of DXA and HR-pQCT scans to investigate associations of phthalate exposure with bone strength and microstructure in cortical and trabecular compartments.

Exposure assessment is the greatest concern in any epidemiologic study measuring phthalate biomarkers. The included studies measured phthalate metabolites in 1-3 spot urine samples. Phthalate metabolites can also be measured in blood, saliva, breast milk, and other biological specimens, but urinary phthalate metabolites have been considered the most feasible and accurate means to assess phthalate exposure [31]. However, due to relatively short biological half-lives of phthalates (3 to 18 hours) and the rapid speed at which they are metabolized and excreted from the body, true exposure to phthalates is difficult to assess [31]. Moderate day-to-day and time-of-day within-person variability in phthalate metabolite concentrations can contribute to misclassification of exposure, especially when only a single spot sample is collected during the study period. Both studies in children performed multiple maternal urinary metabolite measurements during pregnancy, and Reeves et al. were the first to use repeated measurements in postmenopausal women. Although small studies have suggested moderate predictability of phthalate metabolite concentrations over several months based on single urine samples [39], larger studies have found low reproducibility and sensitivity of phthalate metabolites over time, indicating that single spot urine samples are not sufficient to quantify long-term exposure [40].

The importance of assessing phthalate exposure over time, along with accounting for bone growth and loss that naturally occurs in early childhood and late adulthood, respectively, make a prospective study design the most appropriate to evaluate the relationship between phthalate metabolite levels and BMD. A prospective design allows us to assess long-term phthalate exposure more accurately with repeated measurements and accounts for changes in BMD that occur over time. Reeves et al. performed cross-sectional and longitudinal analyses and found different associations between several metabolites and BMD with each approach. Although the explanation for different results is unclear, it emphasizes the importance of assessing changes in BMD longitudinally. Similarly, different associations between maternal urinary phthalate metabolite concentrations and child BMD were found by van Zwol – Janssens et al. at age 6 versus 10 years. Associations between maternal phthalate concentrations and BMD also differed within each trimester of pregnancy. Multiple urine sample collections and BMD scans should be implemented over several time points to account for temporal variability of phthalate metabolite concentrations and BMD.

In each of the included studies, the associations between individual phthalate metabolites and BMD were evaluated. Kuiper et al. were the only to examine this association using phthalate metabolite mixtures in addition to using single biomarker models. Using BKMR and quantile g-computation, they found more pronounced positive associations when assessing mixture models compared to single models. Human exposure to phthalates occurs in complex mixtures from multiple sources, and many phthalates display toxic effects as a group [9]. Assessing phthalate metabolites as mixtures may help to understand their full impact on bone.

Both Min & Min and Reeves et al. observed significant inverse associations between certain phthalate metabolite concentrations and BMD at the third quartile, but not the fourth quartile, compared to the first quartile of phthalate metabolite concentration. However, this type of non-monotonic dose-response (NMDR) relationship, in which the slope of the dose-response curve changes direction within the range of observed doses, is commonly observed with EDCs [41]. Interestingly, associations between phthalate mixtures and BMD estimated by Kuiper et al. were generally positive-linear among females, but nonmonotonic and U-shaped among males. A discussion of these observed sex-dependent associations follows.

The associations between phthalate metabolite level and BMD appeared to vary by sex and by other participant characteristics in different studies. Child’s sex modified the association between MEP, MBzP, and MCPP and BMD in the HOME study, which may be explained by sex differences in levels of estrogen and testosterone, hormones involved in the regulation of bone processes [30]. In older adults, females experience greater bone loss, fractures, and bone disease compared to males, which is attributable to estrogen deficiency that accompanies menopause [1]. Estrogen and other hormones are key regulators of bone homeostasis and it is possible that sex differences are related to the endocrine-disrupting properties of phthalates [42]. Hormone therapy is a common treatment used by postmenopausal women to reduce bone loss and preserve BMD [43]. In studies of postmenopausal women, Min & Min adjusted for use of hormones, but did not evaluate HT use as an effect modifier. Reeves et al. stratified all analyses by HT use and found significant associations between phthalates and BMD among non-HT users, but no associations among HT users. The positive effects of HT use on BMD are well established, so stratification by HT use is recommended in future studies. Large sample sizes are also needed to perform stratification and subgroup analyses by HT use and sex with substantial power.

A number of potential covariates related to phthalate exposure and BMD should be considered in future studies. Risk factors for osteoporosis recognized by the National Institute on Aging include age, family and personal history of fracture, surgical and early menopause, calcium and vitamin D deficiency, physical inactivity, cigarette smoking, and use of certain medications [44]. Additional relevant factors include body mass index (BMI), adiposity, estradiol concentrations, alcohol consumption, caffeine intake, and race/ethnicity [45]. Socioeconomic factors have also been linked to phthalate exposure, and several variables were used to assess socioeconomic status in the included studies [46]. Household income was obtained by Kuiper et al. and DeFlorio-Barker et al., while maternal education level was collected by van Zwol – Janssens et al. and Reeves et al. Kuiper et al. also adjusted for maternal blood lead concentrations. Additional information on diet, use of products, and occupational exposures may be collected to help discern primary sources of phthalate exposure.

All studies used creatinine adjustment methods to account for urinary dilution, although specific approaches varied. Creatinine, a breakdown product from muscle metabolism, is influenced by gender, age, race/ethnicity, diet, physical activity, body composition, and other individual factors [31]. DeFlorio-Barker et al., Min & Min, and Reeves et al. included creatinine as a covariate in multivariable models to account for urine dilution [26–28]. Treating creatinine as a separate covariate in models may lead to less bias in results since creatinine is correlated with several individual factors, and it allows other variables in the model to be independent of the effects of creatinine [47]. Van Zwol-Janssens et al. converted urinary concentrations of phthalate metabolite groups to μmol/g creatinine by dividing average metabolite concentrations over 3 trimesters by average creatinine concentration [29]. Kuiper et al. performed covariate-adjusted standardization in which phthalate metabolite concentrations were multiplied by the ratio of predicted to observed creatinine concentrations using regression models [30]. Recent simulation studies have determined that combined methods of covariate-adjusted standardization and the inclusion of creatinine as a covariate in regression models reduce measurement error bias and better account for day-to-day and person-to-person variations in urine dilution compared to standardization by division approaches [48].

Specific gravity (i.e., the ratio of densities between a urine sample and pure water) is a less expensive method for dilution adjustment compared to creatinine [31], although it is similarly influenced by sociodemographic factors and body composition [31, 49]. In a study comparing dilution adjustment approaches to measure urinary biomarker data, specific gravity-corrected phthalate metabolite concentrations had greater agreement between two approaches (covariate-adjusted standardization and correction using a sample mean of the dilution indicator) compared to creatinine-corrected concentrations. Creatinine- and specific gravity-adjusted concentrations showed slightly higher agreement with each other using the covariate-adjusted standardization method compared to correction using the sample mean of the dilution indicator [49]. Specific gravity correction on urinary phthalate metabolites has also shown higher within-person reproducibility and less systematic variation related to BMI and time of day compared to creatinine in pregnant and postpartum women [47]. Many of the studies used creatinine since it was the only option available, but specific gravity should be considered as an alternative or additional approach for dilution adjustment in future studies.

## Conclusion

Few studies have investigated the association between urinary phthalate metabolite concentrations and BMD in human populations. While existing studies are provocative and suggest negative associations between phthalates and BMD in older adults and positive associations in children, their findings must be considered in light of their methodological limitations, and further investigations are required to confirm their findings. Future studies of phthalates and BMD should utilize repeat measures of the exposure and outcome, assess additional measures of bone health and microarchitecture, and allow for extended follow-up throughout critical periods of the life course. The increasing clinical and economic burden of fractures and osteoporosis indicates a need to elucidate the association between phthalate exposure and BMD.

## Supplementary Information

**Additional file 1.** Systematic approach to critiquing epidemiologic studies.

## Data Availability

Not applicable.

## Abbreviations

BKMR: Bayesian kernel machine regression
aBMD: Areal bone mineral density
BMAD: Bone mineral apparent density
BMD: Bone mineral density
BMI: Body mass index
DBP: Dibutyl phthalate
DEHP: Di(2-ethylhexyl) phthalate
DiBP: Di-isobutyl phthalate
DINP: Diisononyl phthalate
DNOP: Di-n-octyl phthalate
DXA: Dual-energy X-ray absorptiometry
EDC: Endocrine disrupting chemical
HMW: High-molecular-weight
HOME: Health Outcomes and Measures of the Environment
HPLC: High-performance liquid chromatography
HR-pQCT: High-resolution peripheral quantitative computed tomography
HT: Hormone therapy
LMW: Low-molecular-weight
MBP: Mono-n-butyl phthalate
MBzP: Mono-benzyl phthalate
MCMHP: Mono(2-carboxymethylhexyl) phthalate
MCNP: Monocarboxynonyl phthalate
MCOP: Monocarboxyoctyl phthalate
MCPP: Mono(3-carboxypropyl) phthalate
MECPP: Mono(2-ethyl-5-carboxypentyl) phthalate
MEHHP: Mono(2-ethyl-5-hydroxyhexyl) phthalate
MEHP: Mono(2-ethylhexyl) phthalate
MEOHP: Mono(2-ethyl-5-oxohexyl) phthalate
MEP: Monoethyl phthalate
MHBP: Monohydroxybutyl phthalate
MHiBP: Mono-hydroxyisobutyl phthalate
MHP: Mono-n-hexyl phthalate
MiBP: Monoisobutyl phthalate
MMP: Monomethyl phthalate
MnBP: Mono-n-butyl phthalate
NHANES: National Health and Nutrition Examination Survey
NMDR: Non-monotonic dose-response
PBM: Peak bone mass
WHI: Women’s Health Initiative
