# Supplementary material for: Phthalates and bone mineral density: a systematic review
Source: Environ Health. 2022 Nov 12;21:108. doi: 10.1186/s12940-022-00920-5 (PMC9652984; doi:10.1186/s12940-022-00920-5)
Supplement: Supplementary file 1 — Additional file 1. Systematic approach to critiquing epidemiologic studies. [file 12940_2022_920_MOESM1_ESM.docx]

**Systematic Approach to Critiquing Epidemiologic Studies**

**Student Name:** Click or tap here to enter text.

**Full Citation of Journal Article:** Click or tap here to enter text.

| ***Study Purpose and Design*** | |
| --- | --- |
| **Describe (i.e., report just the facts described in the article)** | **Critique (i.e., think through strengths/limitations of the methods and results; address the key questions listed, and add additional comments as needed)** |
| What is the objective/purpose of the study?  Click here to enter text. | Is sufficient justification for the stated objective/purpose provided? Is there a plausible biologic or theoretical mechanism provided?  Click or tap here to enter text. |
| What type of study design was used?  Click or tap here to enter text. | Is this an appropriate design for addressing the stated objective/purpose? Why or Why not?  Click or tap here to enter text. |

| ***Study Population*** | |
| --- | --- |
| **Describe (i.e., report just the facts described in the article)** | **Critique (i.e., think through strengths/limitations of the methods and results; address the key questions listed, and add additional comments as needed)** |
| What is the study base (i.e., the underlying population from which cases were selected?) Be specific—include geographic region, calendar time, age, race, ethnicity, sex, etc.  Click or tap here to enter text. | Is this an appropriate population in which to address the stated objective/purpose? Why or why not?  Click or tap here to enter text. |
| What eligibility criteria were specified?  Click or tap here to enter text. | Are these criteria appropriate? Why or why not?  Click or tap here to enter text. |
| How were participants selected and/or recruited?  Click or tap here to enter text. | - Did method of subject selection differ between comparison groups (i.e., differential loss-to- follow-up in exposed compared to unexposed in a cohort study; differential selection of cases compared to non-cases in a case-control study)?   Click or tap here to enter text.   - Could the study results have been affected by selection bias? Describe direction, magnitude and likelihood.   Click or tap here to enter text.   - To what larger population may the results of this study be generalized (e.g., the study base, additional groups)? If you include groups beyond the study base, justify why. If you think results couldn’t be applied to certain groups, justify why. Were response/participation rates sufficient?   Click or tap here to enter text.   - What other strengths and/or limitations do you note?   Click or tap here to enter text. |
| What is the sample size? (specify total, cases/controls, exposed/unexposed, etc. as appropriate)  Click or tap here to enter text. | Are sample size/power calculations provided? Is there sufficient statistical power for testing the stated hypotheses?  Click or tap here to enter text. |

| ***Exposure Assessment*** | |
| --- | --- |
| **Describe (i.e., report just the facts described in the article)** | **Critique (i.e., think through strengths/limitations of the methods and results; address the key questions listed, and add additional comments as needed)** |
| What was the primary exposure of interest?  Click or tap here to enter text. | Is this exposure appropriate for addressing the stated objective?  Click or tap here to enter text. |
| How was the primary exposure measured? Be specific—include details of measurement tools, assays, etc. used.  Click or tap here to enter text. | - Was the measurement of exposure reliable? Was the measurement of exposure valid? What strengths and/or limitations do you note?   Click or tap here to enter text.   - Could the study results have been affected by **differential** misclassification of exposure? Why or why not? If so, describe direction, magnitude and likelihood.   Click or tap here to enter text.   - Could the study results have been affected by **nondifferential** misclassification of the exposure? Why or why not? If so, describe direction, magnitude and likelihood.   Click or tap here to enter text. |

| ***Outcome Assessment*** | |
| --- | --- |
| **Describe (i.e., report just the facts described in the article)** | **Critique (i.e., think through strengths/limitations of the methods and results; address the key questions listed, and add additional comments as needed)** |
| What was the primary outcome of interest?  Click or tap here to enter text. | Is this outcome appropriate for addressing the stated objective?  Click or tap here to enter text. |
| How was the primary outcome measured? Be specific—include details of measurement tools, assays, etc. used.  Click or tap here to enter text. | - Was the measurement of outcome reliable? Was the measurement of outcome valid? What strengths and/or limitations do you note?   Click or tap here to enter text.   - Could the study results have been affected by **differential** misclassification of outcome? Why or why not? If so, describe direction, magnitude and likelihood.   Click or tap here to enter text.   - Could the study results have been affected by **nondifferential** misclassification of the outcome? Why or why not? If so, describe direction, magnitude and likelihood.   Click or tap here to enter text. |

| ***Statistical Analysis*** | |
| --- | --- |
| **Describe (i.e., report just the facts described in the article)** | **Critique (i.e., think through strengths/limitations of the methods and results; address the key questions listed, and add additional comments as needed)** |
| What statistical analyses were performed?  Click or tap here to enter text. | Is the analytic approach appropriate for testing the specified hypotheses? Why or why not?  Click or tap here to enter text. |
| How was confounding addressed and controlled? (include both approaches in how the study was designed and how data were analyzed) Describe the important confounders that were considered.  Click or tap here to enter text. | Were these approaches sufficient to control for confounding? What is the likelihood of residual confounding? Should the authors have considered the effect of other variables not included in the study?  Click or tap here to enter text. |
| What, if any, effect modification was evaluated?  Click or tap here to enter text. | Were the methods to evaluate effect modification sufficient? Why or why not?  Click or tap here to enter text. |

| ***Results and Conclusions*** | |
| --- | --- |
| What was the major result of the study? Include information on direction and magnitude of association for most important finding. [e.g., Give the actual OR and confidence interval, and also interpret the findings in words. Be sure to mention the comparison category in your text (e.g., compared to individuals who did not drink alcohol)  Click or tap here to enter text. | Was the stability of the result assessed (i.e. did the authors report subgroup and/or stratified analyses, explore alternative exposure and/or outcome definitions, etc?) If so, describe the analyses and their results.  Click or tap here to enter text. |
| What conclusions do the authors make?  Click or tap here to enter text. | - Are these conclusions justified given the results? Why or why not? If not, what conclusions do you feel are appropriate?   Click or tap here to enter text.   - How do these findings compare to those of prior studies? What factors might account for any differences noted?   Click or tap here to enter text. |

| ***Overall Assessment*** |
| --- |
| Are there any potential conflicts of interest? (e.g. funding sources, institutions, etc.) If COIs are noted, how might they affect the reported findings?  Click or tap here to enter text. |
| What are the most important strengths of the study? For each, describe in detail why this is an important strength and how it affects internal and/or external validity.  Click or tap here to enter text. |
| What are the most important limitations of the study? For each, describe in detail why this is an important limitation and how it affects internal and/or external validity? Is the potential effect of the limitation on the results minor or major?  Click or tap here to enter text. |
| How would you characterize the overall quality of the study?  Choose an item.  Justify your choice.  Click or tap here to enter text. |
| In no more than five (5) sentences, summarize the key features and conclusions of the study.  Click or tap here to enter text. |
